# Sense of Coherence in Patients with Inflammatory Bowel Disease

**DOI:** 10.1155/2014/989038

**Published:** 2014-01-02

**Authors:** Randi Opheim, May Solveig Fagermoen, Lars-Petter Jelsness-Jørgensen, Tomm Bernklev, Bjørn Moum

**Affiliations:** ^1^Department of Gastroenterology, Division of Medicine, Oslo University Hospital, P.O. Box 4956, Nydalen, 0424 Oslo, Norway; ^2^The Institute of Clinical Medicine, University of Oslo, P.O. Box 1171, Blindern, 0318 Oslo, Norway; ^3^Department of Nursing Science, Institute of Health and Society, University of Oslo, P.O. Box 1130, Blindern, 0318 Oslo, Norway; ^4^Østfold University College, K.G. Meldahlsvei 9, 1671 Fredrikstad, Norway; ^5^Department of Gastroenterology, Østfold Hospital Trust, 1603 Fredrikstad, Norway; ^6^Research and Development Department, Telemark Hospital Trust, 3710 Skien, Norway

## Abstract

*Background and Aim*. Sense of coherence (SOC) is a health-promoting concept reflecting a person's view of life and response to stressful situations and may be of importance in coping with chronic illness. The aim of this study was to explore associations between SOC and sociodemographic, disease-related, and personal characteristics in a sample of patients with inflammatory bowel disease (IBD). *Methods*. Measures included sociodemographic and disease-related data, the Sense of Coherence Scale, General Self-Efficacy Scale (GSE), and Fatigue Severity Scale (FSS-5). *Results*. In total, 428 IBD patients had evaluable questionnaires (response rate 93%). The overall mean SOC total score was 66.25 (SD 11.47) and with no statistically significant difference between patients with ulcerative colitis (UC) and patients with Crohn's disease (CD). In the multivariate analyses, higher GSE scores were significantly associated with higher SOC scores and higher FSS-5 scores were significantly associated with lower SOC scores in both UC and CD. *Conclusion*. GSE and FSS-5 contributed more to the variance in SOC than sociodemographic and disease-related variables. Longitudinal studies are warranted to investigate the value of SOC as a predictor of disability, medication adherence, coping behavior, and health-related quality of life.

## 1. Introduction

The inflammatory bowel diseases (IBD), Crohn's disease (CD), and ulcerative colitis (UC) are chronic inflammatory disorders of the gastrointestinal tract of unknown etiology. The course of disease is characterized by periods of symptom flares and periods with quiescent disease. Common symptoms are diarrhea, bloody stools, fever, fatigue, and abdominal pain [1–3]. As with many chronic diseases, IBD patients' quality of life and psychosocial function have been shown to be influenced by their disease [4–8]. Further, patients diagnosed with IBD at a young age and with a severe disease course have an increased risk for work disability [9].

Coping with a chronic illness such as IBD involves complex cognitive, physical, emotional, psychological, and behavioral processes [5]. Patients must be able to manage complex medication regimens, find meaning in and adapt to changeable life conditions, and deal with emotions associated with the fact that the disease is not curable. The unpredictable disease course also poses challenges for the patients' daily life as well their life in general [10]. Given the complexity of living with a chronic illness, personal resources may be of importance for patients' well-being, quality of life, and ability to cope with their disease.

The concept “sense of coherence” (SOC) is used to describe a person's capacity to respond to stressful situations, such as chronic illness. The concept was introduced by the medical sociologist Antonovsky in his theory of salutogenesis, which focuses on personal resources needed to move toward and maintain health [11]. SOC consists of a motivational component (meaningfulness), a cognitive component (comprehensibility), and a behavioral component (manageability) [11]. Confronted with a stressor, a person with a high SOC is motivated to cope (meaningfulness) and believes that the challenge is understood (comprehensibility) and that the resources to cope are available (manageability) [11]. Systematic reviews of research on SOC in general populations and in chronic diseases conclude that SOC is strongly related to a person's mental health [12], is associated with health behaviors [13], and has a substantial impact on health-related quality of life (HRQoL) [14]. In addition, SOC is applicable in learning processes and evaluation of education programs [15, 16] and is found to be a predictor for medication adherence [17].

Results from studies of people with other chronic diseases and in the general population indicate that sociodemographic variables, such as age, gender, relationship status, educational level, and work status, may influence SOC [15, 18]. Also, the presence of IBD with its relapsing and remitting disease course may play a role in the person's SOC [19]. However, the relationship between sociodemographic and disease-related variables and SOC has not been adequately studied in IBD patients.

Several studies have identified fatigue as one of the leading concern for IBD patients [20, 21]. A qualitative explorative study found that the IBD patients reduced social and work activities in an attempt to manage fatigue [22]. Individuals who feel they can create environments appropriate to their psychological and physical needs experience less fatigue and fatigue-related stress [23]. Thus, a person's SOC may affect the experience of fatigue interference.

Self-efficacy, defined as a person's beliefs about their capability to perform the behaviors needed to obtain a desired outcome [24], is also found to be important for coping and health behavior. Previous research has shown that persons with high levels of self-efficacy show less reaction to psychological stress and are less prone to depression and anxiety than persons with lower levels of self-efficacy, suggesting a close relation between self-efficacy and coping [25]. Thus, when exploring SOC in IBD patients, the role of self-efficacy is important to consider.

Knowledge and understanding of the salutogenic framework and the SOC concept can help health professionals to understand the IBD patient's health/disease behaviors and choices, to complement clinical data. Because few studies have been undertaken to assess SOC in an IBD population, the aim of the present study was to explore associations between sociodemographic, disease-related, and personal variables and SOC in CD patients and UC patients.

## 2. Materials and Methods

### 2.1. Study Design and Study Population

This descriptive cross-sectional study examined IBD patients who attended outpatient clinics at hospitals in eastern, western, and southern Norway from October 1, 2009, to May 31, 2011. Patients who were ≥18 years with previously verified IBD (clinical, endoscopical, and histological) were included after giving their informed consent. The patients were asked to complete self-administered questionnaires during a regular visit at the hospital but were also given the option to complete the questionnaires at home and return them in a stamped addressed envelope.

### 2.2. Measurements

#### 2.2.1. Sociodemographic Variables

Collected data included age, gender, educational level (12 years (secondary) education or less versus more than 12 years (college/university education)), marital status (married or cohabitant versus single, divorced, or partnered but living separately), work status (working, including being a student versus not working, including pensioner and work disabled), and smoking status (yes (defined as once or more daily) versus no).

#### 2.2.2. Disease-Related Variables

Data concerning medical history and past surgical history for IBD were obtained from the patients' medical records. The Montreal classification was used to classify disease location and behavior in CD and disease extent in UC [26]. Disease activity was measured with the Harvey-Bradshaw activity index (HBAI) in CD patients [27] and with the simple clinical colitis activity index (SCCAI) in UC patients [28]. The patient self-reported disease-related information regarding presence of comorbidities (any of the following: cardiovascular disease, diabetes, arthritis, arthralgia, asthma, dermatological disease, or cancer) and a history of any adverse drug reactions to conventional medications for their IBD (any of the following: nausea, abdominal pain, diarrhea, headache, fever, weight gain, mood changes, joint pain, sleep disturbance, or skin itch).

#### 2.2.3. The Sense of Coherence Scale

To measure SOC, a 13-item short version of the 29-item *Sense of Coherence Scale* was used [11]. The SOC instrument has been translated into 33 languages and studied in 32 countries, thus regarded as applicable to all cultures [15]. The Norwegian version was subjected to forward and backward translation according to recommended procedures [29]. SOC-13 measures the degree to which an individual views the world as comprehensible (5 items), manageable (4 items), and meaningful (4 items) using a 7-point Likert scale. The total SOC score is the sum of the items, ranging from 13 to 91, and the subdimensions ranges 5–35 (comprehensibility) and 4–28 (manageability and meaningfulness). Higher scores reflect a stronger SOC. The SOC-13 scale is reported to be a reliable and valid instrument, with a reported internal consistency (Cronbach's *α*) of 0.70 to 0.92 [15, 30]. Internal consistency in this study was demonstrated with a Cronbach's *α* of 0.86 in the UC group and 0.85 in the CD group, which corresponds well with another Norwegian study [31] and which is considered good [32].

#### 2.2.4. The General Self-Efficacy Scale

GSE [33] measures the strength of an individual's belief in his/her ability to cope with difficult demands in life. It consists of 10 statements that respondents rate on a scale from 1 “completely disagree” to 4 “completely agree.” A GSE scores are calculated by summing each individual score (range 10 to 40). The GSE has been translated into several languages, including Norwegian [34]. The scale has been reported as a reliable and valid instrument [35]. Internal consistency in our study was 0.90 in both the UC and the CD groups, which corresponded well with another Norwegian study [36] and which is considered excellent [32].

#### 2.2.5. The Five-Item Fatigue Severity Scale

FSS-5 was used to assess the experience of fatigue interference with activities of daily living [37]. Each item is scored on a 7-point Likert-type scale ranging from 1 (disagree) to 7 (fully agree). The mean of the five-item scores represents a continuous variable with values from 1.0 (no fatigue interference) to 7.0 (maximum fatigue interference). A higher score indicates higher fatigue interference. The original nine-item version has been validated in the Norwegian general population and in populations with chronic disease and has shown good psychometric properties [38, 39]. However, the five-item version was used in our study because it has been found to improve the psychometric properties of the FSS [37, 40]. In our sample Cronbach's *α* was of 0.90 in the UC group and 0.88 in the CD group, indicating good internal consistency [32].

### 2.3. Statistical Analysis

Differences between groups were assessed with Chi-square (*χ*²) test for categorical data and independent sample *t*-test for continuous variables with normal distribution. When the continuous variables had skewed distributions, they were described with medians and ranges, and differences between groups were tested using the nonparametric Mann-Whitney *U* test. Pearson's correlation coefficient (*r*) was used for bivariate correlation analysis. Multiple linear regression analyses were used to determine the associations between relevant sociodemographic and disease-related variables, and GSE and FSS-5 (independent variables) with SOC and its subdimensions (dependent variables). In addition to gender and age, variables with *P* value < 0.10 in bivariate analyses were entered into a multiple linear regression model. Sociodemographic variables were included in step 1, disease-related variables in step 2, fatigue interference in step 3, and self-efficacy in step 4. The strength of association was determined by the standardized beta coefficient. None of the independent variables included in the final analyses were highly correlated (Pearson's correlation coefficient > 0.7) with any other possible variable; thus we assumed no multicollinearity. Cronbach's *α* [41] was used to assess internal consistency of the scales. Because of multiple testing the level of significance was set to 1% in all analyses. All tests were two sided. Analyses were performed using SPSS for Windows [42].

### 2.4. Ethical Considerations

The Regional Committees for Medical and Health Research Ethics in Norway (reference number: S-00858b) and the internal data protection officer at Oslo University Hospital approved this study. All patients received verbal and written information about the study prior to providing written informed consent.

## 3. Results

Of the 460 patients included in the study after giving informed consent, 30 patients did not return the questionnaire after one reminder. Two patients did not complete the FSS-5, SOC-13, and GSE and were consequently excluded, leaving a representative sample of 428 patients (response rate = 93%). The respondents and nonrespondents were comparable with regard to gender, age, disease duration, and type of diagnosis (data not shown).

Characteristics of the study sample are presented in Table 1. Compared to UC patients (*n* = 190), the CD patients (*n* = 238) were younger (38 versus 41 years, *P* = 0.019) and a higher proportion was female (CD 55% versus UC 43%, *P* = 0.018). In addition, CD patients had significantly longer disease duration (11 years versus 6 years, *P* < 0.001) and a significantly higher proportion had undergone surgery for their IBD compared to the UC group (CD 55% versus UC 7%, *P* < 0.001). Among the UC patients, 55% were classified with extensive disease, whereas 31% of the CD patients had penetrating disease behavior (complications such as fistulas and abscesses) (data not shown).

### 3.1. Distribution of SOC, FSS-5, and GSE

In the sample, SOC total scores ranged from 28 to 91 points, with a mean of 66.25 points (SD = 11.47) and with no gender differences: 66.99 (SD = 11.45) for men and 65.49 (SD = 11.46) for women.  Table 2 presents SOC, FSS-5, and GSE scores in the UC group and the CD group. No significant differences were found between UC and CD with regard to SOC total scores, the SOC subdimensions, or GSE scores (data not shown). The mean FSS-5 score was higher in CD patients 4.49 (SD = 1.48) compared to UC patients 4.13 (SD = 1.54), *P* = 0.015.

In the UC group, men had significantly higher GSE scores compared to women (*P* = 0.003). In the CD group, compared to women, men had significantly higher score on the SOC sub-dimension manageability (*P* = 0.007).

### 3.2. Bivariate and Multivariate Relationships to SOC

Bivariate relationships to SOC and its subdimensions are shown in Table 3. In both disease groups, higher GSE score was associated with higher SOC total and sub-dimension scores, and higher FSS-5 score was associated with lower SOC total and sub-dimension scores. In the UC group, disease activity was negatively associated with comprehensibility whereas, in the CD group, disease activity was negatively associated with manageability.

The linear regression analyses in UC and CD are shown in Tables 4 and 5, respectively. In both disease groups, higher GSE scores were independently associated with higher SOC total scores. The contribution of GSE to the variance of SOC total was similar in both UC and CD. In contrast, higher FSS-5 scores were independently associated with lower SOC total scores. Neither in UC nor in CD, the sociodemographic and disease-related variables were significantly associated with SOC total when controlling for FSS-5 and GSE.

Higher GSE scores were independently associated with higher sub-dimension scores in both the UC and CD groups. In UC, FSS-5 was negatively associated with lower scores at all three subdimensions. In CD, FSS-5 was negatively associated with manageability and meaningfulness. FSS-5 explained more of the total variance of the subdimensions in the UC group compared to the CD group. After controlling for FSS-5 and GSE scores, neither sociodemographic variables nor disease-related variables were associated with SOC sub-dimensional scores.

## 4. Discussion

The aim of this study was to explore associations between sociodemographic and disease-related variables, fatigue interference, self-efficacy, and SOC total and its subdimensions in IBD patients. No significant differences were found in SOC scores between UC and CD patients. The key finding was that self-efficacy had a strong positive association with SOC, whereas fatigue interference had a strong negative association with SOC after controlling for sociodemographic and disease-related variables.

The SOC-13 instrument has been used in a number of studies, including both general and various chronic disease populations [15]. Few studies have investigated SOC in IBD patients [19, 43–46] and only two studies used the SOC-13 scale [43, 44]. We found the mean SOC total score for our IBD patients (66.25) to be comparable with estimates of Swedish IBD patients (69.00) [43] and higher than that in Japanese IBD patients (53.84) [44]. In addition, our results were comparable with estimated mean SOC total score at about 70.00 in the general population [15, 47].

We found, in accordance with other studies, a strong correlation between high self-efficacy and high SOC [31, 48, 49]. Both SOC and self-efficacy have been found to be positively correlated with characteristics such as high self-esteem, internal locus of control, achievement motivation, self-control, optimism, and satisfaction [12, 31, 35]. Hence, SOC and self-efficacy are personal resources important to promote health [11, 24]. According to Antonovsky, SOC is a dispositional orientation; a person with a strong SOC is more likely than a person with a weak SOC to define a stimulus as a nonstressor because he/she is confident that things will work out well [11]. A systematic review supported this hypothesis; the stronger the SOC, the less the symptoms and distress [12]. Self-efficacy influences people's choices and behavior [35] and accordingly may be an important resource needed to strengthen SOC.

The FSS-5 measures the degree to which fatigue interferes with physical functioning and activities of daily living. In the current study, higher FSS-5 scores were found to be negatively associated with SOC. Fatigue has been described elsewhere by IBD patients to impact on thoughts and daily management and to create feelings of “not wanting to bother with anything” [22, 50]. These descriptions indicate that personal resources may impact on management of fatigue in daily living. Fatigue has also been found to have a negative impact on HRQoL and to be related to poor sleep quality, anxiety, depression, and stress in IBD patients [50–53]. These results may indicate that fatigue strongly impacts IBD patients' health and well-being. However, as our study had a cross-sectional design, we were unable to identify the direction of the associations between fatigue interference and SOC. The amount of variance in SOC explained by fatigue interference was higher in the UC group compared to the CD group. In UC, the subdimensions comprehensibility and manageability were most affected by fatigue interference. These dimensions refer to that a situation is perceived as understandable and resources to deal with the situation are available. We could not control for psychosocial factors such as psychological distress and poor sleep quality and cannot exclude that the relationships between SOC and fatigue were confounded with such factors.

Disease activity scores were negatively associated with SOC in the bivariate analyses but not in the multivariate analyses. The unpredictable nature of IBD may be important to consider when assessing the relationship between disease activity and psychosocial factors. Because the symptoms of IBD are fluctuating, a timeframe of the last twenty-four hours in the HBAI and the SCCAI may be a limitation when using cross-sectional data. Further, manifestations such as fecal inconsistency and urgency may reinforce the experience of “uncontrollability” of the disease. These manifestations are found to limit the sense of control over social, working, and personal lives for IBD patients [54]. A negative association between current disease symptoms and HRQoL has previously been reported in IBD patients [6–8, 55, 56]. It has been noted in systematic reviews of other chronic conditions that SOC may serve as a mediator between stressors and HRQoL, with a higher SOC being associated with increased HRQoL scores [14]. Hence, the nature of the relationship between SOC, disease symptoms, and HRQoL needs additional investigation in future longitudinal studies within IBD populations.

We found no significant association between sociodemographic variables and SOC scores after controlling for disease-related variables, fatigue interference, and self-efficacy in our study. Contrary to these results, a systematic review on SOC research concluded that SOC total scores tend to increase with age and that men tend to have a higher SOC score than women [15]. Since the patients were recruited from outpatient clinics at hospitals, our results may not be replicable in a population-based cohort of IBD patients. In the theory of SOC, family life, work, and educational attainment are seen as “resistance resources” that contribute to the development of SOC [11]. In previous studies, the quality of the relationship with partner, quality of paid work, and social support has been found to be strongly associated with SOC [12, 18]. In qualitative interviews, CD patients have expressed that important resources were social support, job satisfaction, and occupational balance [57]. We did not include questions about the quality of the family life and of paid work.

In this study, we also explored the three subdimensions comprehensibility, manageability, and meaningfulness. The bivariate analyses revealed that some of the sociodemographic and disease-related variables were correlated with the SOC subdimensions. Although these associations were not statistically significant in the multivariate analysis, the findings are of interest from a clinical point of view. Antonovsky claimed that the three SOC subdimensions are interrelated and dynamic [11]. This means that a patient with a low SOC total score may have a high score on one or two subdimensions. In clinical setting this is important. Including the patient in the interpretation of the SOC sub-dimension scores where the patient gives meaning to the scores in view of his/her life situation and resources available may lead to that the patients are able to improve an unsatisfactory situation [47, 58]. Studies of chronic diseases have reported significant changes in SOC scores and improved coping as results of interventions based on salutogenic treatment principles [16, 59].

In conclusion, in both UC and CD, higher self-efficacy had a positive association with SOC, whereas fatigue interference was negatively associated with SOC. Longitudinal studies are warranted to investigate the value of SOC as predictor of disability, medication adherence, HRQoL, and coping behavior in IBD patients.

## Figures and Tables

**Table 1:** Characteristics of patients with ulcerative colitis (*n* = 190) and Crohn's disease (*n* = 238).

Characteristics	UC	CD	*P* value
Sociodemographic characteristics			
Age, median (range)	41 (20–79)	38 (18–75)	0.019
Female gender (*n*, %)	82 (43)	130 (55)	0.018
Education > 12 years (*n*, %)	92 (49)	108 (46)	0.558
Work status = working/student (*n*, %)	134 (71)	148 (62)	0.064
Marital status = married/cohabitating (*n*, %)	149 (78)	160 (67)	0.012
Current smoking, yes (*n*, %)	15 (8)	65 (27)	<0.001
Disease-related characteristics			
Disease duration in years, median (range)	6 (0.2–45)	11 (0.1–44)	<0.001
HBAI median (range)		5 (0–30)	
SCCAI median (range)	4 (0–15)		
Previous surgery for IBD (*n*, %)	13 (7)	130 (55)	<0.001
Comorbidities, yes (*n*, %)	84 (45)	88 (37)	0.137
Adverse drug reaction, yes (*n*, %)	107 (57)	142 (60)	0.487

Abbreviations: UC: ulcerative colitis; CD: Crohn's disease; HBAI: Harvey-Bradshaw activity index; SCCAI: simple clinical colitis activity index.

Continuous variables were assessed by the Mann-Whitney *U* test. Chi-squared test (*χ* ^2^) was used to compare proportions.

**Table 2:** SOC, FSS-5, and GSE scores in patients with ulcerative colitis and Crohn's disease.

Ulcerative colitis	All (*n* = 190)	Women (*n* = 82)	Men (*n* = 108)	*P* value*
Total SOC score, mean (SD)	66.74 (11.75)	66.32 (11.51)	67.06 (11.97)	0.665
Comprehensibility	19.33 (3.88)	24.29 (5.36)	24.28 (5.61)	0.985
Manageability	25.58 (5.52)	20.34 (3.70)	20.84 (4.07)	0.383
Meaningfulness	21.83 (4.04)	21.68 (4.05)	21.94 (4.05)	0.660
FSS-5, mean (SD)	4.13 (1.54)	4.37 (1.62)	3.95 (1.45)	0.063
GSE, mean (SD)	30.04 (5.55)	28.67 (5.53)	31.07 (5.37)	0.003

Crohn's disease	All (*n* = 238)	Women (*n* = 130)	Men (*n* = 108)	*P* value*

Total SOC score, mean (SD)	65.85 (11.25)	64.96 (11.45)	66.92 (10.96)	0.182
Comprehensibility	19.30 (3.93)	24.15 (5.26)	25.09 (4.95)	0.160
Manageability	25.48 (5.25)	19.58 (4.05)	20.95 (3.71)	0.007
Meaningfulness	21.07 (4.07)	21.23 (4.06)	20.87 (4.10)	0.498
FSS-5 mean (SD)	4.49 (1.48)	4.68 (1.55)	4.26 (1.36)	0.031
GSE mean (SD)	29.40 (5.44)	29.23 (5.88)	29.61 (4.89)	0.601

Abbreviations: SOC: Sense of Coherence; FSS-5: Five-Item Fatigue Severity Scale; GSE: General Self-Efficacy Scale.

*P values estimated between women and men by independent sample *t*-test.

**Table 3:** Bivariate correlations (Pearson's *r*) between age, disease activity scores, disease duration, fatigue interference, self-efficacy, and SOC in ulcerative colitis and Crohn's disease patients.

	SOC total	Comprehensibility	Manageability	Meaningfulness
Ulcerative colitis (*n* = 190)				
Age in years	0.01	0.02	−0.07	0.06
SCCAI	−0.15	−0.19^a^	−0.13	−0.05
Disease duration in years	0.07	0.11	0.00	0.04
FSS-5	−0.48^b^	−0.50^b^	−0.40^b^	−0.33^b^
GSE	0.51^b^	0.46^b^	0.47^b^	0.41^b^

Crohn's disease (*n* = 238)				
Age in years	0.11	0.11	0.07	0.09
HBAI	−0.17	−0.12	−0.17^a^	−0.14
Disease duration in years	0.09	0.06	0.06	0.11
FSS-5	−0.42^b^	−0.28^b^	−0.36^b^	−0.44^b^
GSE	0.48^b^	0.42^b^	0.38^b^	0.45^b^

Abbreviations: SCCAI: simple clinical colitis activity index; HBAI: Harvey-Bradshaw activity index; FSS-5: Five-Item Fatigue Severity Scale; GSE: General Self-Efficacy Scale.

^a^ *P* < 0.01, ^b^ *P* < 0.001.

**Table 4:** Four independent linear regression analyses with SOC total and subdimension scores as dependent variables in patients with ulcerative colitis (*n* = 190).

Independent variables	SOC total score		Comprehensibility		Manageability		Meaningfulness	
	β	*P* value	*β*	P value	β	P value	β	P value
Step 1.								
Gender (female = 0)	−0.13	0.030	−0.15	0.021	−0.08	0.219	−0.08	0.211
Age	0.00	0.998	−0.01	0.527	−0.06	0.328	0.06	0.388
Education level (≤12 years = 0)			0.07	0.233				
Work status (not working = 0)			−0.01	0.677				
Marital status (not married/cohabitating = 0)	0.14	0.021	0.11	0.100	0.10	0.111	0.16	0.018
Explained variance (*R* ^2^)	**6.2%**		**7.0%**		**4.8%**		**6.2%**	
Step 2.								
SCCAI	−0.02	0.744	−0.06	0.311	−0.02	0.814		
Comorbidities (no = 0)	−0.03	0.638			−0.03	0.624		
*R* ^2^ change	**2.9%**		**2.9%**		**2.2%**		**0.01%**	
Step 3.								
FSS-5	−0.40	<0.001	−0.42	<0.001	−0.33	<0.001	−0.25	<0.001
*R* ^2^ change	**19.0%**		**20.1%**		**13.3%**		**9.3%**	
Step 4.								
GSE	0.44	<0.001	0.39	<0.001	0.39	<0.001	0.36	<0.001
*R* ^2^ change	**16.0%**		**13.0%**		**13.6%**		**11.5%**	

Total *R* ^2^	44.8%		43.0%		33.8%		27.0%	

Abbreviations: *β*: standardized beta coefficient; SCCAI: simple clinical colitis activity index; FSS-5: Five-Item Fatigue Severity Scale; GSE: General Self-Efficacy Scale.

In addition to gender and age, variables that had a Pearson correlation coefficient with *P* < 0.10 in bivariate analyses were included in final regression models.

**Table 5:** Four independent linear regression analyses with SOC total and subdimension scores as dependent variables in patients with Crohn's disease (*n* = 238).

Independent variables	SOC total score		Comprehensibility		Manageability		Meaningfulness	
	β	*P* value	*β*	*P* value	*β*	*P* value	*β*	*P* value
Step 1.								
Gender (female = 0)	0.05	0.425	0.06	0.336	0.14	0.023	−0.09	0.103
Age in years	0.11	0.070	0.11	0.096	0.06	0.359	0.06	0.333
Education level (≤12 years = 0)	0.09	0.144	0.04	0.590	0.13	0.045	0.07	0.251
Work status (not working = 0)					−0.03	0.666	0.01	0.859
Marital status (not married/cohabitating = 0)	0.10	0.090	0.09	0.174	0.14	0.023		
Current smoker (no = 0)	−0.06	0.305			−0.03	0.643	−0.11	0.063
Explained variance (*R* ^2^)	**9.1%**		**4.7%**		**11.3%**		**8.7%**	
Step 2.								
HBAI	0.00	0.990	−0.01	0.866	−0.01	0.856	0.02	0.694
Disease duration in years							0.08	0.186
Adverse drug reaction (no = 0)	−0.08	0.157	−0.11	0.075	−0.10	0.104		
*R* ^2^ change	**3.1%**		**2.3%**		**2.8%**		**2.3%**	
Step 3.								
FSS-5	−0.31	<0.001	−0.17	0.012	−0.26	<0.001	−0.38	<0.001
*R* ^2^ change	**11.2%**		**4.8%**		**7.4%**		**15.3%**	
Step 4.								
GSE	0.42	<0.001	0.39	<0.001	0.31	<0.001	0.36	<0.001
*R* ^2^ change	**15.9%**		**14.2%**		**8.7%**		**11.9%**	

Total *R* ^2^	39.3%		26.0%		30.3%		38.2%	

Abbreviations: *β*: standardized beta coefficient; HBAI: Harvey-Bradshaw activity index; FSS-5: Five-Item Fatigue Severity Scale; GSE: General Self-Efficacy Scale.

In addition to gender and age, variables that had a Pearson correlation coefficient with *P* < 0.10 in bivariate analyses were included in final regression models.
